# Dr. Everly’s Case in the November Number

**Published:** 1887-04

**Authors:** 


### DR. EVERLY'S CASES IN THE NOVEMBER NUMBER.

Dr. Everly writes to us of this now well-known case, saying that he is still unable to relieve the patient. At his request, we publish a more detailed statement of the symptoms in the hope that some one of our readers will be able to suggest the remedy.

Mrs. L. B., aged fifty-six years, was married at sixteen years of age. Had a good constitution, was healthy and happy, but in less than six months her husband told her that women were for men's comfort, and they ought to be satisfied if they got their living. This crushed her feelings. She became filled with a desolate, lonely feeling, almost unendurable.

She gave birth to fifteen children in forty years, raising twelve. She scarcely ever lived in one place a year at a time, always residing on the frontier, subjected to all kinds of hard work and exposure.

When thirty years of age her family moved about four hundred miles, late in the fall, she walking and carrying a child much of the distance. Sharp pains began, shooting through her head and eyeballs, and finally, one night, whilst sewing, a sharp pain went through her head, seeming to burst it to pieces. She was delirious when her husband came to her, lying on the floor with her head clenched between her hands, and screaming that her head was burst. This lasted about eight hours. Very little was done, as no doctor could be obtained. Had frequent attacks after this when overworked or was pregnant, but not so severe as the first. Head very sore afterward. Her hair, which was black, turned very gray inside of three months. Her general health remained quite good until the fall of her forty-fourth year. About this time, her husband being an invalid, she was obliged to give him treatment with a galvanic battery every day for about eight months, by placing a sponge electrode under his feet, and holding the other in her hand, slapping his back from fifteen to thirty minutes at a time; in other ways was working very hard. This seemed to bring on palpitation of the heart, with prolapsus uteri and hemorrhoids, with constant hard pain in splenic region. The menses stopped suddenly. She placed herself under the treatment of an allopath, who gave her sulphate of Morphia, which she took for about six years, constantly increasing the dose, until at last she used one-quarter of an ounce in a week. Washed clothes for a living for about two years, and in washing for a woman with erysipelas she took it in her eyes. In about three weeks after this had granulation of eyelids. The doctor burnt them with caustic and blue stone, turning down the lids and shaving the granules off, and cauterizing until the parts turned white. This was continued for seven or eight months. Quinine was given several times, until she was entirely delirious. These severe spells with her head continuing, she tried steaming, by heating three bricks, wrapping them with wet cloths, and placing one at each side and top, and covering the whole with a large pillow. She remained thus about three-quarters of an hour. Unconsciousness came on and lasted five hours. When consciousness returned her head felt as though it had been melted and all run together, and so terribly sore that the softest pillow felt hard. The spells continued. Was treated about four years by this doctor. The last year had sinking spells, in which while perfectly conscious could not speak or move. The flesh felt like that of a dead person. A new physician was tried. Had a long spell of sickness. Her stomach was so disorganized by drugging that she could not eat any solid food for three months. During this spell she imagined she was in an orchard, when her head came off. She got it in her apron to carry it home, and in climbing the fence let it fall against a tree, and it burst to pieces, but finally getting the pieces home, watched the doctor put it together. But one piece on the right side of the forehead would not fit, so he put it in his pocketbook, and after that in those spells with her head, would say if we put that piece back the pain would stop.

During this spell had all kinds of fancies; saw all kinds of beautiful birds and animals. Was in this condition about six weeks, slowly recovering. She stopped the use of Morphia, but in severe attacks she took Chloral, Chloroform, and Ether. This state continued until two years ago, when the pain went to her heart, causing it to stop beating until apparently dead. About a year ago came under homœopathic treatment. Soon after began having bearing-down pains and misery, as if the menses were coming on. At two or three different times had a discharge of a brownish color. These pains came on every month during her first pregnancy. The veins on the back part of the right thigh burst, and there came a swelling as large as the first, almost preventing her sitting. Had trouble with this whenever pregnant, as the menses came on. After menses ceased had no more trouble with this until these pains came, when the swelling of the vein extended to the knee, with a purple color. At this time has pains beginning in the uterus extending to the hip and knee, with sharp, cutting pains in hypogastric region, a heavy pulling pain in sacral region, hard, dull pain in splenic region, or left hypochondria, shooting up through the heart or left shoulder blade, a drawing, bursting pain in occiput, with fullness and bursting pain in the front part; dry throat at night; bitter taste and foul breath; great soreness through the chest and pit of stomach, more so at night; sharp pains over the whole body; the inside of eyelids dry, the edges smart and burn; a coldness in small of back, as of cold lead; the right limb cold, with cold heels; numbness of the whole body at times; hemorrhoids protruding almost constantly when standing, very painful and sore, gather and break at times.

Aggravation at night from being alone; grief, melancholy, and deep thought; pains in the heart, with smothering; cannot get breath until she gets a drink of cold water and fresh air; is very nervous.

Amelioration, better in open air and from motion, from being in company.

Inside of eyelids is dry, edges smart and burn; coldness of back as if a piece of ice were lying on right side of sacrum; coldness of right leg; cold heels; numbness of whole body at times; hemorrhoids protruding almost constantly; very painful when standing, gathering and discharging at times; feeling of loneliness, although surrounded with friends; feels as if she were in the way of others; despair, does not wish to live; nervous, cannot sleep, especially after five o'clock A. M.; feels as if heart would stop beating, must move to keep it going; smothering sensation, relieved by drinking cold water. General symptoms change almost every day. Is better under my treatment than ever before. When I first saw her the eyes were so inflamed she could not recognize her own children.

---

Sensitiveness of neck in region of tonsils as if the necktie were too tight: Lachesis (Hahnemann *Materia Medica Pura*).

Sensitiveness of larynx to touch and when turning the neck: Spong. (Hering).

